# Supplementary material for: Causal Relationship between Aging and Anorexia Nervosa: A White-Matter-Microstructure-Mediated Mendelian Randomization Analysis
Source: Biomedicines. 2024 Aug 16;12(8):1874. doi: 10.3390/biomedicines12081874 (PMC11351342; doi:10.3390/biomedicines12081874)
Supplement: Supplementary file 1 [file biomedicines-12-01874-s001.zip › Supplementary Figure/Figure S4/Singe SNP plot of MR analysis for TL to ubm-b-1780.pdf]

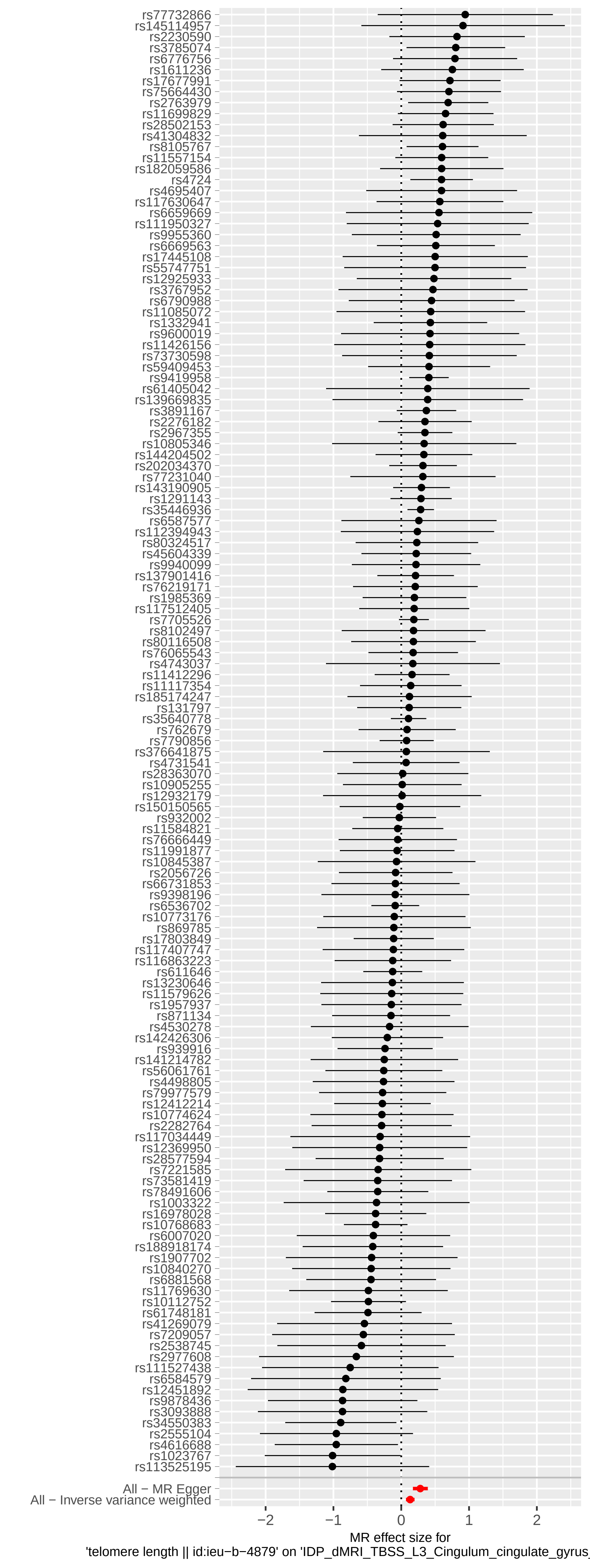
rs77732866
rs145114957
rs2230590
rs3785074
rs6776756
rs1611236
rs17677991
rs75664430
rs2763979
rs11699829
rs28502153
rs41304832
rs8105767
rs11557154
rs182059586
rs4724
rs4695407
rs117630647
rs6659669
rs111950327
rs9955360
rs6669563
rs17445108
rs55747751
rs12925933
rs3767952
rs6790988
rs11085072
rs1332941
rs9600019
rs11426156
rs73730598
rs59409453
rs9419958
rs61405042
rs139669835
rs3891167
rs2276182
rs2967355
rs10805346
rs144204502
rs202034370
rs77231040
rs143190905
rs1291143
rs35446936
rs6587577
rs112394943
rs80324517
rs45604339
rs9940099
rs137901416
rs76219171
rs1985369
rs117512405
rs7705526
rs8102497
rs80116508
rs76065543
rs4743037
rs11412296
rs11117354
rs185174247
rs131797
rs35640778
rs762679
rs7790856
rs376641875
rs4731541
rs28363070
rs10905255
rs12932179
rs150150565
rs932002
rs11584821
rs76666449
rs11991877
rs10845387
rs2056726
rs66731853
rs9398196
rs6536702
rs10773176
rs869785
rs17803849
rs117407747
rs116863223
rs611646
rs13230646
rs11579626
rs1957937
rs871134
rs4530278
rs142426306
rs939916
rs141214782
rs56061761
rs4498805
rs79977579
rs12412214
rs10774624
rs2282764
rs117034449
rs12369950
rs28577594
rs7221585
rs73581419
rs78491606
rs1003322
rs16978028
rs10768683
rs6007020
rs188918174
rs1907702
rs10840270
rs6881568
rs11769630
rs10112752
rs61748181
rs41269079
rs7209057
rs2538745
rs2977608
rs111527438
rs6584579
rs12451892
rs9878436
rs3093888
rs34550383
rs2555104
rs4616688
rs1023767
rs113525195
All − MR Egger
All − Inverse variance weighted
−2
−1
0
1
2
MR effect size for
'telomere length || id:ieu−b−4879' on 'IDP_dMRI_TBSS_L3_Cingulum_cingulate_gyrus_
